# Artificial Affinity Proteins as Ligands of Immunoglobulins

**DOI:** 10.3390/biom5010060

**Published:** 2015-01-30

**Authors:** Barbara Mouratou, Ghislaine Béhar, Frédéric Pecorari

**Affiliations:** 1 INSERM UMR 892 - CRCNA, 8 quai Moncousu, BP 70721, 44007 Nantes Cedex 1, France; 2 CNRS UMR 6299, 8 quai Moncousu, BP 70721, 44007 Nantes Cedex 1, France; 3 University of Nantes, 8 quai Moncousu, BP 70721, 44007 Nantes Cedex 1, France

**Keywords:** immunoglobulin, Fc, alternative scaffold protein, Affibody, Affitin, DARPin, monobody, knottin, CBM

## Abstract

A number of natural proteins are known to have affinity and specificity for immunoglobulins. Some of them are widely used as reagents for detection or capture applications, such as Protein G and Protein A. However, these natural proteins have a defined spectrum of recognition that may not fit specific needs. With the development of combinatorial protein engineering and selection techniques, it has become possible to design artificial affinity proteins with the desired properties. These proteins, termed alternative scaffold proteins, are most often chosen for their stability, ease of engineering and cost-efficient recombinant production in bacteria. In this review, we focus on alternative scaffold proteins for which immunoglobulin binders have been identified and characterized.

## 1. Introduction

During recent decades, the use of peptidic tags has provided an efficient way to detect and purify proteins of interest. However, there are cases, such as therapeutic proteins or monoclonal antibodies, for which the modification of the protein is not desirable or not possible.

Natural bacterial proteins have been identified with affinity and specificity for some immunoglobulin (Ig) families and isotypes (see [1] for a review). Their use has spread widely in laboratories as tools for basic research and in industry for downstream processes of therapeutic antibodies. For example, Protein A from *Staphylococcus aureus* [2] is able to bind human IgG, IgM, IgA, IgE and IgD via interaction with the Fc region. Similarly, Protein L from *Peptostreptococcus magnus* [3] recognizes the five families of Igs although interacting with their light chains. In addition, Protein G from group G *Streptococci* [4] binds human IgG, but not IgM, IgA, IgE and IgD. Thus, the choice of the ligand is critical for the outcome of the targeted application. The major drawback of these natural bacterial Ig binders is that their profile of recognition may not fit specific usages. Furthermore, their use can induce time-consuming and costly engineering work in order to adapt them to the harsh conditions of demanding applications, such as affinity chromatography for which the affinity ligand must resist the extreme pH needed for elution of targets and cleaning of columns [5,6,7,8]. An unstable ligand can leach from columns thereby complicating downstream processes and increasing production costs [9].

**Figure 1:**
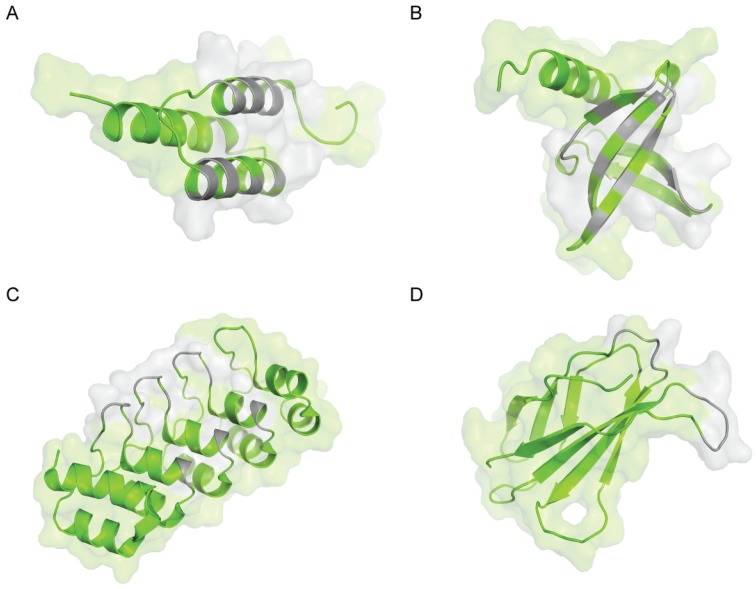
Some structures of molecular basis (shown in green) used to derive artificial binders with examples of associated library designs (shown in grey). (**A**) Synthetic domain Z based on the B domain of Staphylococcal Protein A (PDB code 1Q2N) [12] used to obtain Affibodies; (**B**) Sac7d protein from *Sulfolobus acidocaldarius* (PDB code 1AZP) [13] used to obtain Affitins; (**C**) Designed ankyrin repeat protein (PDB code 1MJ0) [14]; (**D**) Fibronectin type III domain (PDB code 1FNF) [15] used to obtain monobodies. Molecular graphics were generated using PyMOL software (The PyMOL Molecular Graphics System, Version 1.7.1.1, Schrödinger, LLC, New York, NY, USA).

Progress in the fields of molecular biology and protein engineering has led to the emergence of novel classes of tailor-made affinity proteins. A starting protein, termed an alternative scaffold protein, is often chosen to display at least the following characteristics: Small size (<20 kDa), only one polypeptide chain, high stability (thermal, chemical, *etc.*), high recombinant production yields and high solubility. By randomizing a set of chosen residues on the alternative scaffold protein’s surface, large libraries of variants with potentially different specificities can be created *in vitro* (Figure 1). Selection techniques, such as ribosome display [10] or phage display [11], can then be used to isolate from these libraries variants specific for a given target used as bait. With this approach, it is possible to generate artificial ligands with the desired properties.

Many alternative scaffold proteins have been proposed and extensively reviewed [16,17,18,19,20]. Here, we give an overview of the artificial ligands designed to have an affinity for immunoglobulins (Table 1). For the sake of clarity, they are classified according to the alternative scaffold from which they originated. This review focuses on validated non-antibody scaffolds whose usefulness in applications has been demonstrated in several publications.

## 2. Z-domain of Staphylococcal Protein A (Affibody)

The Z-domain of staphylococcal Protein A is one of the most used alternative scaffolds and is the molecular basis of Affibodies. It is derived from the immunoglobulin-binding domain (B-domain) of Protein A, a *Staphylococcus aureus* cell wall protein [21]. The B-domain is a relatively short peptide of 58 amino acids, which is folded into a structure of three α-helices (Figure 1A). It possesses no disulfide bonds and displays reversible folding. The B-domain was early mutated at key positions, mainly for enhanced chemical stability, and the resulting engineered variant, which has a high thermal stability (T*_m_* = 78 °C), was denoted the Z-domain [22]. In 1995, first-generation Affibody libraries were created by randomization of 13 solvent-accessible residues in helices 1 and 2, including many (but not all) positions critical for IgG recognition [23]. Initially, phage display technology was used to identify library members that bind to various targets; more recently, ribosome display has also been used [24]. Affibodies with dissociation constants (K_D_) in the nanomolar [25] and picomolar [26] ranges have been reported. Although their production requires a denaturation/refolding procedure, the structures of several Affibodies have been determined alone or in complex with their respective target, showing that the three α-helix bundle is conserved [27,28]. Recently, the design of an optimized Affibody sequence was described with improved thermal (T*_m_* = 69 °C *vs.* 65 °C) and storage stability, reduced residual interaction with immunoglobulins, higher hydrophilicity, and greater suitability for peptide synthesis [29]. The use of Affibodies has been demonstrated for a number of biotechnological, diagnostic and therapeutic applications (for a review, see [30]). In a recent work, Affibodies with affinity for neonatal Fc receptor were shown to extend half-life of recombinant proteins in mice [31]. Moreover, Affibodies have been identified with specificity for different immunoglobulins, such as IgA, IgE and IgG.

Affibodies showing human IgA binding were selected against two different human IgA_1_ monoclonal antibodies as target molecules [32]. Five Affibody variants were capable of IgA binding, with dissociation constants (K_D_) in the range between 0.5 and 3 μM. The variant Z_IgA1_ with the strongest binding affinity was further analyzed and shown to recognize both human IgA subclasses (IgA_1_ and IgA_2_) as well as secretory IgA with similar efficiencies. This gives Z_IgA1_ an IgA binding profile closer to those of the Arp4 and Sir22 receptin proteins isolated from *Streptococcus pyogenes*, which recognize the CH_2_-CH_3_ interface on the IgA Fc fragment. No detectable cross-reactivity towards human IgG, IgM, IgD or IgE was observed. In addition, the affinity chromatographic recovery of IgA from a bacterial lysate and unconditioned human plasma was demonstrated.

Affibody molecules with human IgE-binding activity have also been isolated with a K_D_ of 0.4 μM [33]. These Affibodies, in monomeric or dimeric forms, have been successfully expressed on the cell surface of *Staphylococcus carnosus*, thus creating a staphylococcal bacterium capable of binding human IgE.

**Table 1:** Summary of alternative scaffolds used to derive artificial binders with Ig specificities.

Scaffold Name	Acronym	Size (aa)	Origin	Mutated Region	Disulfide Bridge	Ig specificity	K_D_ (nM)	References
Z-domain of staphylococcal Protein A	Affibody	58	Bacteria (*Staphylococcus aureus*)	13 aa on two α-helices	No	Human IgA	500–3000	[24,32,33]
						Human IgE	400	
						Mouse IgG_1_	1.8–1300	
Archeal “7 kDa DNA binder” protein family	Affitin	~65	Archaea (*Sulfolobus acidocaldarius*, *Sulfolobus solfataricus*)	10-14 aa on a β-sheet surface, and on two loops	No	Human IgG_1,2,4_	34–74	[34,35,36,37]
						Human IgG_1–4_	400–5280	
						Mouse IgG	26	
						Chicken IgY	30	
						Rabbit IgG	2638	
Carbohydrate-binding module	CBM	168	Bacteria (*Rhodothermus marinus*)	12 aa at the binding site	No	Human IgG_4_	N.D.	[38]
Designed ankyrin repeat protein	DARPin	67 + (n × 33)	Artificial sequence from a consensus analysis	7 aa on a β-turn and on an α-helix of repeats	No	Human IgG_1_	2.1–137	[39,40,41]
						Human IgE	0.9–6.8	
Cystine-knot miniprotein	knottin (Min-23)	23	Plant (*Ecballium elaterium*)	10 random aa inserted	Yes	Several monoclonal antibodies	15.5	[42]
Fibronectin type III domain	monobody	94	Human	Various number of aa randomized in three loops	No	Goat IgG	1.2–35	[43,44]
						Bovin IgG	N.D.	
						Rabbit IgG	0.051–1.08	
						Mouse IgG	4.1	

N.D.: Not determined.

Murine IgG_1_ specific binders were obtained from a combinatorial ribosome display library of Affibody molecules K_D_ in the low nanomolar to low micromolar range [24]. The use of three different mouse IgG_1_ monoclonal antibodies as alternating targets resulted in the identification of binders with broad mouse monoclonal IgG_1_ recognition. Interestingly, the most thoroughly investigated variant Z_mab25_ showed a narrow binding preference for mouse IgG_1_ in contrast to the widely used Proteins A and G. Mapping of the binding site for the Z_mab25_ Affibody indicated that an epitope on the CH_1_ domain of mouse IgG_1_ Fab was recognized, overlapping with the Protein G-binding site. The narrow specificity of Z_mab25_ enabled the recovery of mouse IgG_1_ from samples resembling a supernatant from hybridoma cultured in medium containing fetal bovine serum.

## 3. Archeal “7 kDa DNA Binder” Protein Family (Affitin, Commercial Name: Nanofitin)

Some biotechnological applications require affinity reagents resistant to harsh conditions. Extremophilic Archaea are microorganisms that have developed an arsenal of proteins able to resist unusual conditions of growth, such as extreme pH, temperature, ionic strength or pressure. Archaea include Sulfolobus, Acidianus, and Metallospharea genera, which host proteins from the “7 kDa DNA binder” family for which no mesophilic equivalents are known. The biological role of these chromatin-like proteins is to prevent melting of the genomic DNA at the high growth temperature of the microorganisms (about 80 °C). We originally described the use of these proteins as alternative scaffolds for Sac7d from *Sulfolobus acidocaldarius* in 2007 [45] and for proteins from other Archaea, such as Sso7d from *Sulfolobus solfataricus*, in 2008 [46] to obtain affinity reagents called Affitins [47]. Sac7d and Sso7d are proteins with a simple molecular organization, one polypeptide chain that lacks cysteine, and are about 20 times smaller than antibodies (7 *vs.* 150 kDa). They both fold as an SH3-like five-stranded incomplete β-barrel capped by a C-terminal α-helix (Figure 1B). They have been shown to be stable from pH 0 up to 12 for Sac7d [37] and up to 13 for Sso7d [48], while their thermal stability has been reported to be as high as 90.4 °C for Sac7d [49] and 100.2 °C for Sso7d [50]. Different library schemes of Sac7d and Sso7d variants have been reported, involving randomization of 10 to 14 amino acids at the surface originally interacting with double-stranded DNA in the wild-type protein. A novel generation of Affitins has been reported that includes an artificially randomized extended loop in addition to the variegated surface [51]. Using these libraries and selection techniques such as ribosome, mRNA or yeast display, binders specific for various targets have been shown to inherit largely the advantageous properties of their parental proteins [34,35,37,45,51,52]. These binders can show affinities for their cognate targets with dissociation constants in the picomolar range [45]. A study reporting the first structure of an Affitin, with an affinity for human IgG, showed that the overall fold of the Sac7d protein was not significantly modified by the randomization process [37]. Recently, the structural basis of two of their modes of binding was validated by solving the structures of three Affitins in complex with their cognate targets [51]. Affitins with recombinant production yields of up to 200 mg/L of *E. coli* flask culture have been reported [45]. Variants derived from Sac7d and Sso7d have been shown to be thermally stable up to 89.6 °C and 102.8 °C, respectively [34,45] with high chemical resistance (pH, chaotropic agents) [34,37,47]. Several applications have been reported: *In vivo* inhibition [45], enzymatic inhibition [51], immunoreagents for western blots and ELISA [45,53], protein chip arrays [54], biosensors [55] and virus purification [52]. Using this family of archeal proteins, Ig binders were obtained.

Variants derived from Sso7d were selected against mouse IgG and chicken IgY, and showed affinities of 26 and 30 nM, respectively [34]. The specificity analysis for mouse IgG selected variant confirmed that it does not recognize chicken IgY, goat IgG, rabbit IgG or the Fc region of human IgG. In another study, a Sso7d variant with a dissociation constant of about 400 nM was obtained by yeast display selection against the human IgG Fc region [35]. According to flow cytometry assays, this binder does not interact with mouse IgG, chicken IgY, rabbit IgG, goat IgG, donkey IgG, Fab or Fab2. These IgG ligands were shown to bind a different epitope on human Fc from that of Protein A, recognizing the CH3 region. Western blot experiments revealed that it was able to recognize all human IgG isotypes as well as the deglycosylated form of human IgG. Using histidine scanning or random mutagenesis, binders with affinities for human IgG in the micromolar range (1.6 and 5.3 μM) at pH 7.4 were also obtained, which displayed a much weaker affinity at pH 4.5. The authors concluded that these binders have the potential to be used for affinity chromatography applications for which elutions under mild pH conditions are desirable to avoid denaturation of the IgG to be purified (usually IgGs are eluted from Protein A columns at pH ~2.7). An Sso7d mutant has also been isolated with specificity for rabbit IgG and a dissociation constant of 2.6 μM [36].

Binders specific for IgG have been isolated by ribosome display from Sac7d libraries using the human Fc region as a target. A first study reported the sequences of several Affitins with affinity for human IgG [37]. Two of them were characterized (C3 and D1), which were representative of two library mutagenesis schemes involving randomized flat surfaces of Sac7d either with or without the use of randomized loops. Their affinities were determined as 74 and 34 nM. According to ELISA experiments, they were both specific to human IgG (isotypes 1, 2, and 4) and did not show interactions with goat, mouse, rabbit, rat, and sheep IgG. While C3 showed an interaction with pig IgG, this was not observed for the Affitin D1. Competition experiments suggested that the epitope recognized on human IgG by the Affitin C3 overlaps with that of Protein A. D1 was shown to recognize a different epitope on human IgG from that of Protein A and of CD64, demonstrating that Affitins can bind the same target via different epitopes. The pH stability of these two binders was investigated: C3 is stable from pH 0 up to 11 and D1 from pH 0 up to 12. Recently, the pH stability of the most pH stable binder was further improved by coupling the grafting of its IgG-binding site from Sac7d to Sso7d to a rational directed mutagenesis approach [48]. The resulting mutant was stable (from pH 0 up to at least 13; up to 76.9 °C), with an affinity for human IgG of 4 μM. This high alkaline resistance should be interesting for the design of reusable affinity columns for antibody purification able to resist harsh cleaning-in-place procedures involving steps with concentrated sodium hydroxide (>0.1 M).

## 4. Carbohydrate-Binding Module (CBM)

The CBM4-2 of the *Rhodothermus marinus* xylanase Xyn10A, a protein module with a molecular weight of 18 kDa, has been developed as a diversity-carrying alternative scaffold. This module displays the type B topology of the binding site (a binding cleft with affinity for free single carbohydrate chains, such as xylans and β-glucans) and is highly thermostable (T*_m_* = 89.3 °C), although it has no disulfide bridges [56]. A small combinatorial library (1.6 × 10^6^ clones) was created by introducing restricted variation at 12 residues around the carbohydrate-binding site [38]. Variants specific towards the human IgG_4_ carrying a λ light chain were successfully selected for, using the phage display method. Investigated clones had completely lost their ability to bind the original substrate, xylan, showed a high productivity when expressed in *Escherichia coli* (up to 111 mg/L of flask culture) and kept the thermostability (T*_m_* = 76 up to 80 °C) of their parental molecule. However, further studies demonstrated that IgG_4_-specific CBMs recognize a protein and not a carbohydrate structure of the target IgG_4_-molecule used and ruled out recognition of the conserved structures found in the constant part of IgG4 λ [57]. Furthermore, K_D_s were not determined but preliminary studies demonstrated rapid binding kinetics of the IgG_4_-specific CBMs with their target.

## 5. Designed Ankyrin Repeat Protein (DARPin)

Ankyrin repeat proteins are naturally found in the bacterial, archeal and eukaryotic kingdoms. These proteins are involved in key protein-protein interactions, such as those occurring in transcription, regulation, and transport [58]. Ankyrin repeat proteins are composed of structurally homologous motifs of about 20–40 amino acids forming a β-turn followed by two anti-parallel α-helices and a loop; two of these repeats form the flanking N- and C-terminal caps (Figure 1C). The number of repeats can differ from one ankyrin to another, suggesting a natural adaptation of their size and binding surface to targets. This observation was exploited to design artificial ankyrin repeat proteins named DARPins (for Designed Ankyrin Repeat Proteins). Combinatorial libraries of DARPins were originally described in 2003 [59]. They are based on a consensus sequence determined by a structure/sequence analysis that is randomized at seven positions of the 33 amino acids composing a repeat unit. Another level of diversification was introduced in the design of these libraries by varying the number of repeats from four to six. Recently, a novel generation of DARPins was reported, termed LoopDARPins, which include an additional randomized loop [60]. Since 2004, a number of DARPins have been isolated by ribosome display or phage display from these libraries. These proteins can show affinities for their cognate targets with dissociation constants in the picomolar range [61]. They usually show high recombinant production yields from *Escherichia coli* cultures (up to 200 mg/L in flask) and high thermal (resistant to boiling) and chemical (up to 5 M guanidinium chloride) stabilities. The first three-dimensional structure of an unselected DARPin has been reported in 2003, showing a fold similar to that of natural ankyrin repeat proteins [59], while the first structure of a DARPin in complex with a target was reported in 2004, thereby validating the mode of binding of these artificial proteins [62]. DARPins have been used for various applications such as: *in vitro* and *in vivo* enzymatic inhibition [63,64], immunohistochemistry [65], biosensors [55], tumor targeting with radiolabeled DARPins [66,67] or with immunotoxins [68], and viral retargeting [69]. Several DARPins have also been isolated against Igs.

Steiner *et al.* described the generation of DARPins with specificity for IgG_1_ [39]. In this work, they used the Fc region of a human IgG_1_ to perform selections by phage display. Four isolated DARPins were characterized and shown to be monomeric and able to bind full-length human IgG_1_ according to ELISA experiments. These binders did not exhibit interactions with mouse IgG_1_ or mouse IgG_2b_. Nanomolar affinities of these binders for the Fc region of human IgG_1_ were estimated, with K_D_ ranging from 2.1 nM to 137 nM.

With the aim of proposing a new drug format for the treatment of severe allergic asthma, DARPins with specificity for human IgE were isolated. In a first study [40], four DARPins were shown to recognize three different IgEs while they did not interact with IgG_1_, IgG_2_, IgG_3_, IgG_4_, IgA_1_, and IgM. Affinities for IgE of two monomeric and two bivalent DARPins were estimated in the low nanomolar range (K_D_ = 0.9 to 6.8 nM). An epitope mapping study showed that DARPins from two sequence families were able to recognize two different epitopes on IgE. Their antagonist activity against the interaction of IgEs with their receptor FcεRI compared well with that of omalizumab, a humanized monoclonal anti-IgE antibody used for the treatment of allergic asthma. In a second study [41], another DARPin (E53) specific for IgE was characterized. This work mainly focused on the characterization of a DARPin-Fc fusion, which was shown to bind free IgE as well as IgE bound to its receptor and FcγRIIb via the Fc region, thus triggering aggregation of FcεRI and FcγRIIb. The fusion was not anaphylatogenic and was able to inhibit allergen-induced basophil activation. The authors concluded that this anti-IgE DARPin-derived molecule might represent a drug candidate.

## 6. Cystine-Knot Miniprotein (Knottin)

Cystine-knot miniproteins, also known as knottins, share a common disulfide-bonded framework, and contain loops of variable length and composition that are constrained to a core of anti-parallel β-strands. This structure confers high thermal (above 95 °C), chemical (guanidine hydrochloride, urea, acidic pH), and proteolytic stabilities. Polypeptides containing cystine-knot motifs are found in proteins with very diverse origins and functions, such as protease inhibition [70,71].

In 1999, a rational design approach focused on downsizing the trypsin inhibitor *Ecballium elaterium* (EETI-II) by N-terminal truncation resulted in the development of a new alternative scaffold named Min-23. This alternative scaffold, containing the cystine-stabilized β-sheets (CSB) motif and lacking the inhibitory loop, comprises only two disulfide bridges instead of the three found in the parental protein. Min-23 was shown to fold in a native-like manner and to have high stability (T*_m_* ~ 100 °C) [72]. A phage library of 2.8 × 10^8^ clones has been constructed by the insertion of 10 random amino acids between residues 16 and 20 of Min-23 within its exposed second β-turn [42]. The selection of this library on a variety of different targets, including several mAbs, led to the isolation of new specific binders. As an example, S42E6 binds to the immobilized anti-EBV antibody (anti-capsid protein gp 125 of Epstein Barr Virus, clone L2) with a K_D_ of 1.55 × 10^−8^ M but does not bind to an immobilized unrelated antibody.

## 7. Fibronectin Type III Domain (Monobody, Commercial Name: Adnectin)

The 10th human fibronectin type III domain (^10^Fn3) was chosen as an alternative scaffold due to its structural similarity to antibody variable domains and was first described in 1998 [73]. Fn3 is a small monomer (94 amino acids, 10 kDa) based on a β-sandwich fold comprised of seven strands connected by six loops (Figure 1D). It is devoid of cysteine and has high thermostability with a melting temperature above 80 °C. The BC, DE, and FG loops of Fn3, which are structurally analogous to the complementarity-determining regions of antibodies, were used to generate diversity by randomizing 10 to 21 positions using different mutagenesis schemes [73,74]. An alternative library, using a randomized β-sheet surface in addition to randomized loops, was used with success to isolate binders [75]. Monobodies have been obtained with dissociation constants in the low or sub-nanomolar range, using affinity maturation when necessary to increase affinities further [74,76]. Various targets were reached by using phage, yeast, mRNA display or yeast-two hybrid techniques [77]. The first crystal structure of a monobody in complex with its cognate target was obtained in 2007 [78].

Direct competition of full diversity (all 20 amino acids allowed) and tyrosine/serine (an attempt to reduce theoretical sequence space enabling isolation of functional clones) diversity libraries was found to be dominated by a full diversity library for the selection of high-affinity binders to goat and rabbit IgG [44]. The highest-affinity goat IgG binder, gI2.5.3T88I, has a K_D_ of 1.2 nM. However, it also binds bovine IgG as well as mouse IgG. Moreover, it has a midpoint of thermal denaturation of 63.9 °C.

The highest-affinity rabbit IgG binder, rI4.5.5, exhibits 51 pM affinity. This binder has a unique specificity for rabbit IgG, with no detectable binding to bovine, chicken, goat, human or mouse IgG, and a midpoint of thermal denaturation of 49.1 °C. Both goat and rabbit highest-affinity biotinylated binders immobilized on a streptavidin agarose column have demonstrated their utility in affinity purification of IgG from serum. Fluorophore-Fn3 conjugates were used as detection reagents in flow cytometry.

In addition, a new more complex library, named “G4”, incorporating selective wild-type conservation at structurally important positions and tailored diversity, has been demonstrated to be superior to the previous libraries used. This library has enabled the generation of binders to a multitude of targets, including mouse IgG [43]. The mI2.2.1 anti-mouse IgG binder has a K_D_ affinity of 4.1 nM. Interestingly, this clone has four cysteines in identical and/or adjacent loops, suggesting feasible disulfide bonding that can stabilize the binder.

## 8. Computationally-Designed IgG-Binding Protein

Computational design provides the opportunity to program protein–protein interactions for desired applications [79]. In this two-step approach, termed *de novo* protein interface design, a set of key residues constituting the epitope to be recognized on the target are identified and the ideal corresponding core interaction residues are computed. In a second step, a large set of alternative scaffold proteins is scanned for surfaces compatible with the presentation of the core residues allowing the formation of interactions with the target epitope.

In 2014, Baker and colleagues used this approach to generate a pH-dependent Fc domain-binding protein based on the pyrazinamidase from the *Pyrococcus horikoshii* alternative scaffold [80]. The optimized FcB6.1 protein binds IgG with a Kd of ~4 nM at pH 8.2, and approximately 500-fold more weakly at pH 5.5. FcB6.1 presents a helix for binding to the “consensus” region between the CH2 and CH3 domain of the Fc, and thus burying the accessible histidine 433 residue. Competition experiments revealed that FcB6.1 interacts in the same region as Protein A and other well-characterized binders.

Additionally, the FcB6.1 protein is extremely stable, heat-resistant (up to 80 °C) and highly expressed in bacteria. It binds tightly to human IgG_1_, IgG_2_, and IgG_4_, and to mouse IgG_1_ and IgG_2a_. FcB6.1 binds only weakly to IgG_3_ (to which Protein A does not bind) and does not bind to rat IgG_2a_. This computationally-designed protein enables the pH-based control of binding for IgG affinity purification.

## 9. Other Alternative Scaffolds

Several other proteins have been used as alternative scaffolds to derive immunoglobulin binders. However, their characterizations were limited (no K_D_ determination or no indication of specie or isotype for Ig recognized). For instance, Tendamistat [81] is a 74 amino-acid β-sheet protein from which several loop mutants with affinity for a monoclonal antibody were selected. The loop including residues 60 to 65 was identified as the main contributor to Ig recognition. Insect defensin A is a 4 kDa protein that folds as an α-helix followed by an anti-parallel β-structure connected by two disulfide bridges. A selection performed with phage display using a library corresponding to randomization of two loops allowed enrichment for insect defensin A mutants with affinity for the anti-BMP-2 monoclonal antibody [82]. However, no sequences were described. The TEM-1 β-lactamase is an enzyme in which randomized peptides were introduced in solvent exposed loops surrounding the active site [83]. Affinities of binders for prostate specific antigen (PSA)-monoclonal antibodies were estimated in the nanomolar to micromolar range (K_D_ = 7.6 nM and 2.1 μM). Zinc finger motifs of the Cys_2_-His_2_ type are composed of about 25 residues that adopt a ββα structure. This domain is stabilized by a zinc ion, which is complexed by two cysteines and two histidines. Mutants with affinity for a monoclonal IgA were isolated from libraries built by randomizing five positions in the α-helical region of a consensus zinc-finger motif [84]. Thioredoxin from *Escherichia coli* is a 108 amino-acid protein, which has been used to build a library of variants by insertion of 12 randomized residues in the active-site loop [85]. Using this library and bacterial display, mutants with affinity for three murine monoclonal IgGs were isolated.

## 10. Conclusions

The generation of artificial binders with specificity and affinity for Igs is now readily achievable using a wide variety of alternative scaffold proteins. Most of these binders inherit the favorable properties of their parent molecule chosen to be suitable for demanding applications. However, a number of these Ig binders have been generated as proof of concept to establish alternative scaffold proteins and are often not as extensively characterized for their specificity as Protein A or Protein G, for instance.

The first generation of alternative scaffold designs has been shown to reach different epitopes on constant regions of Igs (DARPins, Affitins, for example). Since 2012, a second generation of monobodies, Affitins and DARPins has emerged, which involves flat surfaces in combination with loop(s) to generate binding activity while conserving the properties of the initial alternative scaffolds. Structural studies of these monobodies and Affitins bound to their cognate target (no Ig binders yet) have highlighted their propensity to bind cleft-shaped epitopes [51,75]. It is probable that in a near future immunoglobulin binders will be developed from this novel generation of alternative scaffold designs.

Computational approaches seem promising to design usable artificial affinity proteins, as exemplified by the work of Strauch *et al.* [80], and could emerge as a fast means to generate Ig binders with the desired properties. Their true potential could become clearer once a number of other examples have been provided.

In summary, the range of tools available to detect or capture Igs is widening, and thus artificial ligands, with their remarkable properties, should be considered in addition to natural ones for a given application.
